# The Use of Fungi of the *Trichoderma* Genus in Anaerobic Digestion: A Review

**DOI:** 10.3390/ijms242417576

**Published:** 2023-12-17

**Authors:** Adrianna Kubiak, Agnieszka A. Pilarska, Agnieszka Wolna-Maruwka, Alicja Niewiadomska, Katarzyna Panasiewicz

**Affiliations:** 1 Department of Soil Science and Microbiology, Poznań University of Life Sciences, Szydłowska 50, 60-656 Poznań, Poland; adrianna_kubiak@interia.pl (A.K.); amaruwka@up.poznan.pl (A.W.-M.); alicja.niewiadomska@up.poznan.pl (A.N.); 2 Department of Hydraulic and Sanitary Engineering, Poznań University of Life Sciences, Piątkowska 94A, 60-649 Poznań, Poland; 3 Department of Agronomy, Poznań University of Life Sciences, Dojazd 11, 60-632 Poznań, Poland; katarzyna.panasiewicz@up.poznan.pl

**Keywords:** anaerobic digestion, *Trichoderma* sp., biogas, biomethane, lignocellulosic biomass, waste plant biomass, pre-treatment methods, biological pre-treatment methods, digestate, organic carrier

## Abstract

Plant waste biomass is the most abundant renewable energy resource on Earth. The main problem with utilising this biomass in anaerobic digestion is the long and costly stage of degrading its complex structure into simple compounds. One of the promising solutions to this problem is the application of fungi of the *Trichoderma* genus, which show a high capacity to produce hydrolytic enzymes capable of degrading lignocellulosic biomass before anaerobic digestion. This article discusses the structure of plant waste biomass and the problems resulting from its structure in the digestion process. It presents the methods of pre-treatment of lignocellulose with a particular focus on biological solutions. Based on the latest research findings, key parameters related to the application of *Trichoderma* sp. as a pre-treatment method are discussed. In addition, the possibility of using the digestate from agricultural biogas plants as a carrier for the multiplication of the *Trichoderma* sp. fungi, which are widely used in many industries, is discussed.

## 1. Introduction

One of the major challenges of a rapidly developing society is ensuring that all people in the world have regular access to sufficient usable energy. This energy is a key aspect of everyday life because it allows us to meet basic human needs and develop individual branches of the global economy. Therefore, with population growth, urbanisation and technological and economic progress, the demand for usable energy will continue to grow exponentially [1,2]. According to Bharathiraja et al. [3], by 2050 global energy demand will increase by at least 50%.

Alalwan et al. [4] report that more than 88% of the usable energy available today is produced from fossil fuels (hard coal, brown coal, crude oil and natural gas). Unfortunately, despite the fact that the above-mentioned sources constitute the basis of the energy system, it turns out that their further exploitation is associated with environmental and economic problems. Fossil fuels are non-renewable sources that are exploited much faster than they are replenished. Global reserves of these minerals are being depleted very quickly, disturbing the balance between supply and demand and leading to higher energy costs. In addition, many years of research have shown that the exploitation of fossil fuels generates high emissions of contaminants, particularly in the form of carbon dioxide and sulphur compounds, which are responsible for climate change and global warming [5,6,7], as well as adversely affect human and animal health [8,9]. It is estimated that in the European Union, the consumption of energy from non-renewable sources generates greenhouse gas emissions of at least 75% [10].

Reports of the depletion of fossil fuel resources and adverse environmental impacts have forced the introduction of regulations in many countries aimed at protecting the natural ecosystem through the use of renewable energy sources (light energy, wind energy, hydropower, geothermal energy and biomass) [11]. Renewable energy is sourced from natural resources that renew themselves continuously in a sustainable manner and do not emit contaminants [7,12,13]. According to the data of the Eurostat, only 21.8% of the energy consumed in the European Union comes from natural resources [14]. In order to reduce the risk of climate change, the European Commission has adopted a regulation aiming at increasing the use of renewable sources up to at least 42.5% by 2030, which is expected to improve the Union’s energy efficiency by 11.7% [15]. Similarly, the United Nations has assumed that energy production from renewable sources should be doubled by 2030 [2]. Moreover, in June 2022, the Environment Council and the European Parliament ordered carbon dioxide emissions into the atmosphere to be reduced by at least 62% by 2030 compared to average emissions in 2005 [16]. The regulations introduced in recent years are intended to bring European Union countries closer to achieving the goal adopted in 2019, which assumes that Europe will be the first continent in the world with zero net greenhouse gas emissions by 2050 [10,17]. In addition, Europe’s achievement of climate neutrality will contribute to the goal adopted in 2016 in the form of the Paris Agreement, which aims to hold the increase in global average temperature to no more than 1.5 °C [18].

In fact, despite it being true that light energy and wind energy are capable of generating large amounts of relatively cheap usable energy, they are not continuous and regular resources. The effectiveness of the above-mentioned sources depends largely on weather conditions and the season. The lack of sun or wind means that much less usable energy is generated [7,19,20]. In addition, a major technical challenge is the storage of surplus light and wind energy. Many years of research have shown that an ideal alternative to the problem in question, irrespective of weather conditions, is the production of energy in the form of biogas from plant waste biomass [21,22,23]. Majeed et al. [2] estimate that bioenergy generated from biomass is capable of meeting almost 40% of global energy demand.

Plant waste biomass generated during agri-food, agricultural, forestry and household production is cheap and is the most abundant renewable energy resource on Earth [20,24,25]. Organic biomass is a neutral source in terms of carbon dioxide emissions because the carbon dioxide generated in the combustion process is necessary for the functioning of plants, which are then used to produce plant waste biomass [3,13,26]. Moreover, plant biomass, which is waste, is used as a substrate for energy production. Thus, plant biomass does not compete with plants intended for the food sector [22,27].

The most common method of utilising organic lignocellulosic mass is anaerobic digestion [28]. Anaerobic digestion is an anaerobic degradation process of organic matter that involves the following four stages: hydrolysis (degradation of complex compounds into simpler forms), acidogenesis (formation of carboxylic acids), acetogenesis (formation of acetate) and methanogenesis (formation of methane) [29,30,31]. It is a highly complex process carried out by anaerobes, in which the products of one stage constitute the substrate for the next [32,33]. As a result of digestion, a mixture of gases, which is called biogas, is produced. Despite the fact that the final composition of biogas depends on many factors (for example, substrate type and properties, process conditions and type of installation) [34,35,36], it is estimated that the mixture consists of 55–70% methane, 30–45% carbon dioxide and, to a lesser extent, hydrogen sulphide, water vapour, nitrogen, oxygen and trace elements [25,30,37]. Biogas and biomethane are highly efficient sources not only of electricity and heat but can also be used as biofuel in automotive motors [20,22,38,39].

The key stage of the anaerobic digestion process, which affects its efficiency and the amount of gases produced, is the rate of substrate hydrolysis [40,41]. Plant biomass is mainly composed of cellulose, hemicellulose and lignin. This complex lignocellulosic structure protects the plant cell wall from degradation by microorganisms and hydrolytic enzymes, which consequently reduces the efficiency of energy production [42,43]. Shrestha et al. [24] report that the resistance of plant biomass to hydrolysis often results in biogas and biomethane generation efficiencies of less than 60% of the theoretical value. Therefore, the main challenge is to develop a pre-treatment method for the substrate that will accelerate its degradation [44,45]. Many years of research have shown that the ideal solution to this problem is the use of fungal strains of the *Trichoderma* genus [46,47,48].

*Trichoderma* sp. is a genus of microscopic filamentous fungi, currently comprising more than 375 species of microorganisms. They are classified as cosmopolitan microorganisms that occur in all soil types and climatic zones worldwide. In addition, they are saprophytes, showing the ability to colonise and degrade dead organic matter [49,50]. Some species of the *Trichoderma* sp. genus reproduce sexually by producing ascospores, which then form fruiting bodies (teleomorph form), while other species reproduce asexually by conidia (anamorph form) [51,52]. Moreover, these microorganisms produce thick-walled spores called chlamydospores, which help them survive unfavourable environmental conditions [53]. The main characteristics of fungi of the *Trichoderma* genus that influence their key use in the anaerobic digestion process are their very strong cellulolytic and hemicellulolytic properties, which allow them to accelerate the hydrolysis of the polysaccharides that make up the cell wall of plant waste biomass. These microorganisms also produce lignin-modifying enzymes responsible for the partial degradation of this component, thus reducing its inhibitory effect on the activity of microorganisms [46,47,48].

What is more, fungi of the *Trichoderma* genus show the ability to multiply on many organic carriers. Many years of research have shown that the ideal substrate for the growth and functioning of these microorganisms is the digestate from agricultural biogas plants, which is a by-product (waste) of the anaerobic digestion process [54,55]. The resulting digestate pulp is characterised by a high content of microelements and macroelements easily assimilated by crop plants, which are essential for their proper growth and functioning. In addition, the components of the digestate have a positive impact on the physico-chemical properties of the soil and are free of pathogens. Properly tested digestate is an ideal alternative to mineral fertilizers [20,56].

The aim of this article is to present and analyse the possibilities of using cosmopolitan fungi of the *Trichoderma* genus in the anaerobic digestion process. This article presents, based on the latest knowledge in the field and the latest research, a perspective on the application of the microorganisms in question in the production of usable energy from a renewable source such as plant waste biomass. Furthermore, attention is drawn to the possibility of using the digestate as a carrier for the multiplication of microorganisms that may constitute the basis of biological plant protection agents and biological agents for stimulating the growth and proper functioning of crop plants. This article contains recommendations on how to improve the efficiency of energy production and waste management, such as plant biomass and digestate from biogas plants.

## 2. The Use of *Trichoderma* Fungi in the Pre-Treatment of Lignocellulosic Biomass

### 2.1. Structure and Composition of Lignocellulosic Biomass

Lignocellulosic biomass is the most commonly generated waste in agricultural, agri-food, forestry and household production [22]. According to Paul and Dutta [57], the resulting by-products can be classified as agricultural and forestry residues, as well as grass, energy crops and woody biomass. In addition, the above-mentioned authors estimate the annual global production of lignocellulosic waste biomass to be 181.5 billion tonnes.

The main components of plant waste biomass are cellulose, hemicellulose and lignin [58], the percentages of which vary highly between plant species [57,59]. Furthermore, the composition of the substrate in question varies according to the age of the plant and its stage of growth [22,60], environmental conditions [23], cultivation method and harvest season [25,61]. In addition to the three main biopolymers, biomass also contains lipids, proteins, pectins, carbohydrates (mainly glucose, sucrose and fructose) [23], extracts and ashes in its structure [44,60].

The basic structural component of the plant cell wall is cellulose [43,44]. This polysaccharide occurs in a linear form, forming straight chains of volumes ranging from 100 to 140,000 units [42]. The structural unit of cellulose is cellobiose, composed of two D-glucose subunits that are bonded to each other by β-1,4-glycosidic bonds [40,46]. The long-chain cellulose polymers then bond together by hydrogen bonds and Van der Waals forces, thus forming higher-order structures called microfibrils [60], which are subunits of macrofibrils [57]. The different orientations of the cellulose molecules give the polymer two degrees of crystallinity [40]. The vast majority of the plant wall component in question occurs in a compact crystalline form, which is resistant to the hydrolysis process. However, a small amount of it takes an amorphous form, which is susceptible to enzymatic degradation and digestion [43,57]. Therefore, the higher the cellulose crystallinity index, the longer the lignocellulosic biomass hydrolysis stage [62,63]. Xu et al. [43] state that the crystallinity index of plant biomass is in the range of 30–80%.

Another structural element of the plant cell wall is hemicellulose, which comprises a heterogeneous group of polysaccharides and their derivatives [43,64]. According to Agregan et al. [44] and Zhou et al. [65], hemicelluloses include, among others, xylans, mannans, xyloglucans, β-(1,3);(1,4)-glucans and galactans. In contrast, Abraham et al. [42] indicate that the plant biomass component in question occurs in an amorphous form with a lower degree of polymerisation than cellulose [66], making hemicellulose more susceptible to the hydrolysis process than other plant cell wall components [62]. Moreover, another property that facilitates the degradation of hemicellulose is its low molecular weight compared to cellulose and the presence of relatively short side chains [60]. The primary function of this component is to increase the degree of mechanical strength of the biomass cell wall [43] through hydrogen bonds with cellulose and covalent bonds with lignin [23,40,63].

The third basic plant component is lignin, described as the most complex fraction of lignocellulosic biomass [44,62,67]. It is a heterogeneous polymer composed of three phenylpropanol molecules [22,43], with a very high degree of crystallinity [25]. The component in question fills available spaces in the plant cell wall [22] and binds through covalent bonds to its other components, thus forming a complex and multistage structure with high resistance to stress factors as well as microbiological and enzymatic hydrolysis [43,44,68]. Moreover, lignin makes the lignocellulosic complex hydrophobic, which significantly reduces the ability of plant biomass to dissolve in water [22]. Bajpai [60] reports that the component in question can also initiate the non-specific adsorption of hydrolytic enzymes, and, in addition, lignin derivatives can be highly toxic to microorganisms carrying out biomass degradation and digestion.

Thus, the high resistance of the lignin fraction to microbiological and enzymatic hydrolysis, the high level of crystallinity of cellulose, and the low percentage of available cellulose surface on which cellulolytic enzymes actively act are the main reasons for the relatively rare use of plant lignocellulosic biomass as a substrate in anaerobic digestion [43,57,58]. Therefore, the main challenge is to develop an effective pre-treatment method for waste biomass that will accelerate the rate of its degradation [44,45,69].

### 2.2. Methods of Pre-Treatment of Lignocellulosic Biomass

Due to the very high resistance of plant waste biomass, pre-treatment, which includes the appropriate preparation of the raw material, is an essential element in the process of producing renewable energy from lignocellulosic biomass [60,70]. The main purpose of this stage is to degrade the lignin–polysaccharide bonds that occur between the different components of the plant cell wall [25,71]. Separating hydrolysis-resistant lignin from the remaining elements will open the matrix and increase the surface area of the raw material available to hydrolytic enzymes and microorganisms, where they can actively act and degrade biomass [23,62,72].

**Figure 1:**
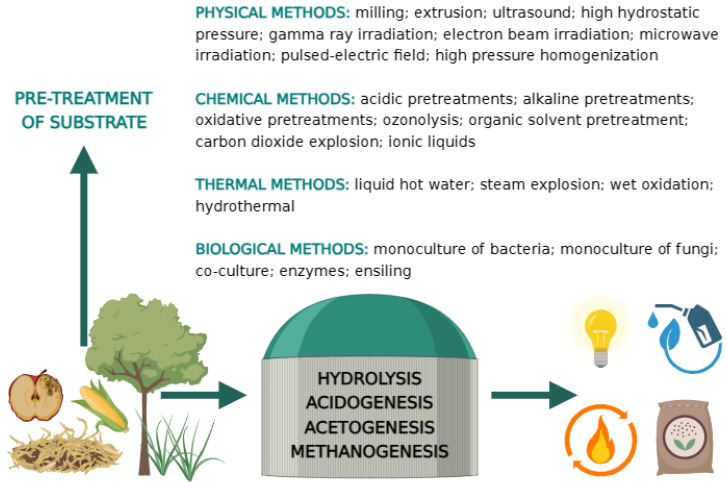
Methods of pre-treatment of lignocellulosic biomass.

To increase the digestibility of plant waste biomass in anaerobic digestion, several pre-treatment methods have previously been developed for the substrate in question, which can be divided into physical, thermal, chemical and biological solutions [26,63,70] (Figure 1, Table 1).

The selection of a suitable and effective pre-treatment method for lignocellulosic raw material depends primarily on the type, composition [70] and physico-chemical properties of the biomass used. The solution used should not have a harmful effect on the environment or human health and should not lead to the production of inhibitors that can inhibit the power generation process [25]. Moreover, the ideal method should be economically viable [62], simple to carry out and fast. Additionally, the desired result is maximum carbohydrate recovery and minimal consumption of chemicals, energy and water [40,70].

Despite their high effectiveness, physical, thermal and chemical pre-treatment methods generate several problems that adversely affect the economic aspect of the entire anaerobic digestion process. The main disadvantage of the above-mentioned solutions is the high cost of the equipment required for the treatment stage. The high demand for energy, water and reagents in the case of chemical solutions leads to a significant increase in the overall costs of using a given method. In addition, chemicals are used to carry out chemical pre-treatment of the raw material, which, by reacting with components of the plant biomass, can generate by-products that are harmful and dangerous for the environment and human health, sometimes acting as inhibitors of the biogas production process [25,40,62,70].

An ideal alternative that meets the requirements when selecting a suitable method of pre-treatment of plant lignocellulosic biomass is the use of microorganisms in the form of single cultures or a consortium of various species of microorganisms, as well as the hydrolytic enzymes produced by them [40,42]. These biological solutions are, above all, safe and environmentally friendly because they involve the use of bacteria and fungi, which are permanent elements of the natural ecosystem. The methods in question do not require the purchase of expensive chemical reagents or specialised equipment or large amounts of energy and water. The microorganisms used to degrade plant waste biomass show the ability to grow and function properly under mild conditions and do not generate harmful by-products that can accumulate in the environment and pose a threat to the natural ecosystem and human and animal health. However, despite many advantages, biological solutions are often described as time-consuming methods because the stage of pre-treatment using microorganisms can last several days or more, which in turn leads to the extension of the entire digestion process. This disadvantage results from the fact that the isolates need time to acclimate to the new environment and then multiply and produce appropriate hydrolytic enzymes. Moreover, another challenge is to create appropriate environmental conditions that will ensure the proper growth and development of the microorganisms, as well as the activity of enzymes that decompose lignocellulosic biomass. Therefore, the key challenge in the use of biological pre-treatment methods is the selection of the appropriate strain or strains of microorganisms, as well as the conditions under which the hydrolysis of the substrate is carried out [23,25,62,70].

### 2.3. Application of Trichoderma Fungi in the Pre-Treatment of Lignocellulosic Biomass

The biological pre-treatment of plant waste matter involves the use of a wide range of hydrolytic enzymes, especially cellulases and hemicellulases. These enzymes are responsible for degrading the individual components of lignocellulosic biomass by creating pores and crevices in their structures through which hydrolytic enzymes migrate. This leads to damage to the lignin fibres, exposure of the secondary cell wall and an increase in the surface available for the action of enzymes and microorganisms [89,90,91,92], which in turn leads to the acceleration of the entire digestion process and an increase in the amount of biogas produced (Table 2). The tools used during biological pre-treatment are single cultures of microorganisms [46,47,90,91,92,93], which have hydrolytic properties, consortia of microorganisms consisting of various microorganisms [48] and hydrolytic enzymes in free or extract form [26,63,70,94].

Mutschlechner et al. [93] indicate that an important parameter determining the degree of increasing the efficiency of biogas production is the amount of *Trichoderma* sp. fungal inoculum used during treatment. The above-mentioned authors proved that as the inoculum density increases, the amount of energy generated increases, but only up to a certain point. Compared to the control, the greatest improvement in process efficiency was obtained when 25 g of the starting substrate was inoculated with *T. viride* spores at 2 × 10^8^. In contrast, increasing the inoculum to 4 × 10^8^ spores resulted in a significant decrease in the efficiency of biogas and biomethane production. Furthermore, it was noted that cellulolytic activity (CMCase activity) was highest at 2 × 10^8^ spores of *T. viride*, whereas at an inoculum density of 4 × 10^8^ the activity was relatively lower. Therefore, based on the results obtained, it was concluded that too many metabolically active microorganisms led to the consumption of all available nutrients, resulting in a reduction in the activity of the cellulase system and substrate degradation efficiency. Similarly, Wagner et al. [46] showed that with increasing inoculum density, the amount of energy generated increases. The amount of biogas and biomethane produced was greater for 15 g of biomass (biogas: 150.19 mL; biomethane 47.36 mL) of *T. viride* than for 5 g of biomass (biogas: 100.79 mL; biomethane 23.42 mL).

Another important parameter is the duration of pre-treatment. A study conducted by Mutschlechner et al. [93] shows that biogas and biomethane yields increased during the first ten days of incubation. In contrast, extending the treatment time by a further ten days led to an increase in energy in control samples, while in samples inoculated with *T. viride* there was a decrease in yield. Regardless of the experimental variant, the three-day incubation ended with very low biomethane production, which was probably due to the acidic reaction of the environment, which led to unfavourable conditions for further stages of the process. In addition, it was found that low pH during pre-treatment can lead to enzyme denaturation and low activity of the cellulase system, the optimum of action of which is 6.5. However, after three days of incubation, the pH began to increase rapidly, mainly due to the degradation of organic acids and ammonia production, leading to the formation of an optimal environment for cellulolytic enzymes. After ten days of treatment, there was a renewed decline in process efficiency due to a decrease in enzyme activity, a decrease in substrate availability, and the accumulation of by-products that disturb the cellulase system. In contrast, Mustafa et al. [89] indicate that the pre-treatment of lignocellulosic biomass with *T. reesei* resulted in the maximum degradation of dry matter, cellulose and hemicellulose at an incubation time of 30 days, while lignin degradation was greatest at 20 days of treatment.

The type and composition of the substrate used in pre-treatment and anaerobic digestion also affect the efficiency of energy production. Kovacs et al. [91] report that the highest amount of methane was obtained when the substrates for *T. reesei* were corn stover, wheat straw and willow chips, respectively. In contrast, based on the results of their own study, Mutschlechner et al. [93] found that the higher the wood content in the substrate, the more pronounced the decrease in biomethane yield. The above-mentioned authors suggest that wood-based materials have a natural acidity, thus leading to a lower pH and creating suboptimal conditions for the functioning of the cellulase system, and contain relatively little protein and nitrogen, which are needed for the activity of hydrolytic enzymes.

Another important parameter is the water content in the substrate. A study conducted by Mustafa et al. [89] shows that, irrespective of the incubation time, the biomethane yield was highest when the moisture content was 75%. On the other hand, at 65% and 85% of the moisture content, the efficiency of the process was lower. Based on these observations, it was concluded that too high a water content limits the amount of oxygen and the growth of the *Trichoderma* fungi, while an optimum moisture level leads to softening of the substrate, swelling of the cellulose present in crystalline form and a reduction in the integrity of the biomass, thus increasing the surface area available for the action of hydrolytic enzymes. Similarly, Mutschlechner et al. [93] showed that the highest efficiency of biogas and biomethane production from water hyacinth occurred at 70% of the moisture content, while at 90% and 50% the energy yield was lower. In addition, the authors report that the *Trichoderma* sp. fungi are hydrophilic microorganisms and prefer substrates with a relatively high water content, which positively influences the activity of their cellulase system.

## 3. The Use of Digestate as an Organic Carrier for the Multiplication of *Trichoderma* Fungi

### 3.1. Structure and Composition of Digestate

The digestate, also known as digestate pulp, is, in addition to biogas, the main product produced in a biogas plant. It is very often referred to as a waste or by-product, which contains undigested residues from the raw materials used in anaerobic digestion, microorganisms and intermediate products of the energy production process [95]. The digestate pulp also contains a wide range of minerals [56] and bioactive substances resulting from the activity of microorganisms, such as vitamins, phytohormones, nucleic acids or monosaccharides, which have a positive effect on the growth and functioning of crop plants [56]. In addition, pathogens and heavy metals such as zinc and copper may be present in the digestate, which pose a risk to human and animal health as well as the environment. Therefore, a key aspect in the further use of the pulp is its thorough testing, in addition to its appropriate treatment into a harmless and safe product [95]. The most commonly used digestate treatment methods include filtration, drying, dilution, membrane technology, flocculation, ion exchange and solid–liquid separation [56].

The digestate pulp is generally separated using mechanical methods into two fractions that are different in physico-chemical terms. A relatively low content of dry matter and organic matter characterises the liquid fraction, while a high content of these elements characterises the solid fraction [56]. The liquid fraction contains a large amount of potassium and nitrogen (70–80% of nitrogen present in the total pulp volume), while the solid fraction contains mainly phosphorus (55–65% of phosphorus present in the total pulp volume) [96]. These nutrients are present in mineralised forms that are directly available and assimilable by crop plants [97]. Furthermore, the amount of nitrogen, phosphorus and potassium in the digestate is equivalent to the weight of these elements in the starting raw material [96]. Lamolinara et al. [98] estimate that the digestate pulp can account for up to 95% of the total mass introduced into the bioreactor at the beginning of the digestion process.

**Figure 2:**
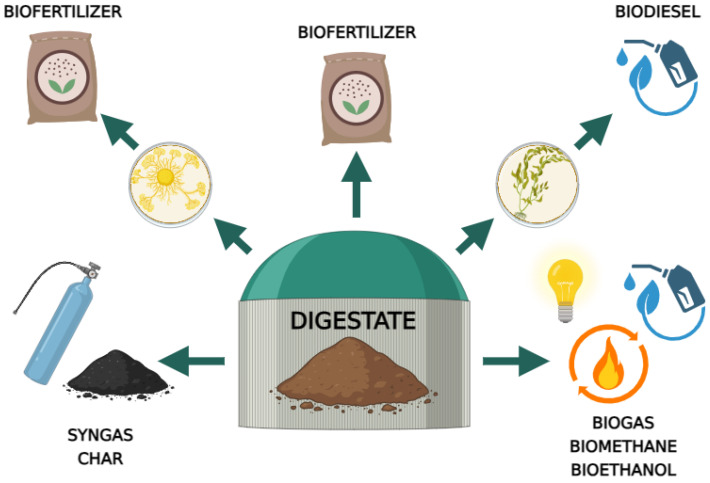
Application of the digestate produced in an agricultural biogas plant.

Therefore, properly tested and prepared digestate is widely used as a natural biofertilizer that provides microelements (boron, chlorine, zinc, manganese, copper, molybdenum, nickel and iron) and macroelements (nitrogen, phosphorus, magnesium, potassium, sulphur and calcium) necessary for the proper growth and functioning of plants [96,99,100,101]. Consequently, digestate pulp is a promising alternative to artificial fertilizers [95,102]. Furthermore, Monlau et al. [97] and Wang and Lee [103] report that the liquid fraction of the digestate can be further used in the biodiesel production process as a substrate for microalgae cultivation. Lamolinara et al. [98] report that the digestate pulp can also be used as a cheap organic carrier for the multiplication of microorganisms with desired properties, which have been widely used in industry. The solid fraction, on the other hand, can be used in biological processes to produce bioethanol, biogas and biomethane, in thermo-chemical transformations to produce syngas and pyrocarbon, and in the composting process [56] (Figure 2).

Monlau et al. [97] and Logan and Visvanathan [96] indicate that the composition of the digestate pulp depends on the type of raw material and inoculum of microorganisms used in the process, the anaerobic digestion conditions, the type of plant, the pre-treatment method used for the substrate and the treatment method of the resulting digestate. Therefore, it is not possible to define universal values for individual parameters that characterise the digestate pulp. However, the above-mentioned authors only report that the reduction in volatile fatty acids that occurs during anaerobic digestion, as well as the production of ammonia and the use of strong alkalis, make the pH of the digestate alkaline above 7.

### 3.2. Application of Trichoderma Fungi to the Digestate

The fungi of the *Trichoderma* genus have a wide range of applications in many different industries. In particular, these microorganisms are an essential component of biological agents for plant protection and plant growth stimulation [53,104]. The relatively high demand for the biomass of the microorganisms in question makes scientists and entrepreneurs look for new and, above all, low-cost microbiological substrates on which large-scale cultivation of the *Trichoderma* fungi can be carried out. The ideal solution to this problem is to use an organic carrier in the form of digestate. This carrier is rich in the nutrients necessary for the proper growth of microorganisms. In addition, the use of digestate pulp will significantly reduce the costs associated with the cultivation of microorganisms, as this digestate is a waste that is generated in very large quantities in agricultural biogas plants.

In their study, Bulgari et al. [55] showed that *T. reesei* and *T. atroviride* achieved maximum mycelial growth after six and three days of solid-state fermentation, respectively, in which the substrate was a mixture of digestate pulp and expired fruit. Moreover, it was found that cultivation on waste did not lead to the loss of the properties responsible for promoting plant growth by the analysed microorganisms. Similarly, Alias et al. [54] demonstrated that *T. atroviride*, *T. reesei*, *T. asperellum* and *T. harzianum* were capable of multiplying on a carrier that consisted of dried digestate and food waste. Additionally, it was observed that the application of digestate enriched with the above-mentioned fungi species led to the elongation of the roots of *Lepidium sativum*. In turn, Bulgari et al. [105] analysed the activity of esterase produced by *T. asperellum* during its cultivation in solid-state fermentation. Based on the results obtained, the authors concluded that the highest activity of esterase was achieved when the substrate consisted of 50% of the digestate and 50% of the food residues enriched with sawdust. In contrast, the results of the study conducted by Escamilla-Alvarado et al. [106] showed that the production of holocellulases by *T. reesei* reached its highest values when the digestate from hydrogenogenic digestion was the carrier for the multiplication of microorganisms.

## 4. Conclusions

The use of plant waste biomass in the anaerobic digestion process to produce renewable energy in the form of biogas and biomethane has enormous economic and environmental potential. From an economic point of view, available pre-treatment methods that aim to increase the efficiency of the entire process are often not practical and cost-effective. A promising solution to this problem is the use of the *Trichoderma* fungi as a biological treatment method. These microorganisms are an integral element of the natural ecosystem and therefore do not pose a threat to it, increasing the ecological value of the entire anaerobic digestion process. In addition, these fungi show a high range of tolerance to stressful and changing environmental conditions, allowing them to grow and function properly on different types of substrates as well as in the environment in which the pre-treatment of lignocellulosic biomass is carried out. Furthermore, the cultivation and multiplication of these microorganisms do not require specialised equipment or large amounts of energy and water. An ideal alternative microbial substrate for the growth of the *Trichoderma* sp. fungi is the use of the waste from the biogas plant, i.e., the digestate, which is rich in nutrients. This process is able to significantly reduce the costs associated with the industrial use of the microorganisms in question.

Unfortunately, despite reports on the possibilities of using fungi of the *Trichoderma* genus in the anaerobic digestion process, the sector of this type of study is still quite limited. The key aspects that should constitute the basis for subsequent reports consist of a thorough analysis and determination of the main parameters, such as the species of fungi used, the concentration of their inoculum, the duration of pre-treatment, and the type, composition and moisture content of the substrate in the form of lignocellulosic biomass or the digestate.

In conclusion, the use of the *Trichoderma* fungi as biological tools in the pre-treatment of lignocellulosic waste substrate is a promising solution to accelerate the hydrolysis of plant biomass as well as the whole process of biogas and biomethane production. The use of digestate as a substrate for the multiplication of the microorganisms in question will reduce the costs associated with the production of these fungi for industrial use. In addition, the combination of the digestate and *Trichoderma* sp. strains with mechanisms to stimulate plant growth and development or to eliminate plant pathogens will produce a comprehensive biopreparation that will have a positive effect on the soil and crop plants. Therefore, the application of the microorganisms in question makes it possible to solve major challenges in terms of increasing the amount of renewable energy generated, using the waste generated in the form of lignocellulosic biomass and digestate, obtaining an innovative biofertilizer and increasing food production.

## Figures and Tables

**Table 1:** Yield of biogas and biomethane in anaerobic digestion using pre-treatment methods [mL/g VS—amount of biogas/methane produced per unit volatile solid at moment; mL/g TS—amount of biogas/methane produced per unit total solid at moment].

Pre-Treatment Methods		Biogas or Biomethane Yield	References
Physical methods	milling	methane yield: 378.75 mL/g VS methane yield in control: 303 mL/g VS	[73]
	ultrasound	biogas yield: 396 mL/g VS biogas yield in control: 139 mL/g VS	[74]
	microwave irradiation	methane yield: 221 mL/g TS methane yield in control: 137.18 mL/g TS	[75]
	high hydrostatic pressure	methane yield: 77.9 mL/g TS methane yield in control: 31.8 mL/g TS	[76]
Chemical methods	potassium hydroxide	methane yield: 258 mL/g VS methane yield in control: 184 mL/g VS	[77]
	sulfuric acid	biogas yield: 424.3 mL/g VS biogas yield in control: 183.32 mL/g VS	[78]
	ethanol	methane yield: 155.4 mL/g VS methane yield in control: 75.3 mL/g VS	[79]
	ozonolysis	methane yield: 432.7 mL/g VS methane yield in control: 260 mL/g VS	[80]
Thermal methods	steam explosion	methane yield: 589 mL/g VS methane yield in control: 366 mL/g VS	[81]
	advanced wet oxidation	methane yield: 289.2 mL/g VS methane yield in control: 220 mL/g VS	[82]
	liquid hot water	methane yield: 202.81 mL/g VS methane yield in control: 124.51 mL/g VS	[83]
	hydrothermal	methane yield: 248.2 mL/g VS methane yield in control: 183.85 mL/g VS	[84]
Biological methods	*Bacillus subtilis*	methane yield: 270.8 mL/g VS methane yield in control: 230.7 mL/g VS	[85]
	*Ceriporiopsis subvermispora*	methane yield: 44.6 L/kg VS methane yield in control: 20 L/kg VS	[86]
	consortium of thermophilic microorganisms	methane yield: 325.7 mL/g VS methane yield in control: 273.7 mL/g VS	[87]
	endoglucanase, xylanase and pectinase	biogas yield: 765.5 mL/g VS biogas yield in control: 529.1 mL/g VS methane yield: 465.4 mL/g VS methane yield in control: 295.2 mL/g VS	[88]

**Table 2:** Yield of biogas and biomethane in anaerobic digestion using *Trichoderma* sp. as biological pre-treatment method [mL/g VS—amount of biogas/methane produced per unit volatile solid at moment; mL/g TS—amount of biogas/methane produced per unit total solid at moment].

Species of *Trichoderma*	Biogas or Biomethane Yield	References
*Trichoderma atroviride*	biogas yield: 223.4 mL/g VS biogas yield in control: 135 mL/g VS methane yield: 200 mL/g VS methane yield in control: 91.84 mL/g VS	[92]
*Trichoderma viride*	biogas yield: 703.7 mL/g VS biogas yield in control: 379.5 mL/g VS methane yield: 356.1 mL/g VS methane yield in control: 194.4 mL/g VS	[93]
*Trichoderma viride*	biogas yield: 790 mL/g VS biogas yield in control: 553.7 mL/g VS methane yield: 447.7 mL/g VS methane yield in control: 314.12 mL/g VS	[93]
*Trichoderma viride*	biogas yield: 840.9 mL/g VS biogas yield in control: 367.4 mL/g VS methane yield: 439.5 mL/g VS methane yield in control: 133.3 mL/g VS	[93]
*Trichoderma viride*	biogas yield: 1299.4 mL/g VS biogas yield in control: 688.3 mL/g VS methane yield: 722.6 mL/g VS methane yield in control: 312.3 mL/g VS	[93]
*Trichoderma viride*	methane yield: 419.63 mL/g TS methane yield in control: 389.13 mL/g TS	[94]
*Trichoderma viride*	biogas yield: 100.79 mL/g VS biogas yield in control: 66.16 mL/g VS methane yield: 23.42 mL/g VS methane yield in control: 11.41 mL/g VS	[46]
*Trichoderma viride*	biogas yield: 150.19 mL/g VS biogas yield in control: 66.16 mL/g VS methane yield: 47.36 mL/g VS methane yield in control: 11.41 mL/g VS	[46]
*Trichoderma reesei*	methane yield: 91.6 NmL/g TS methane yield in control: 9.4 NmL/g TS	[90]
*Trichoderma reesei*	methane yield: 90.1 NmL/g TS methane yield in control: 9.2 NmL/g TS	[90]
*Trichoderma reesei*	methane yield: 94.3 NmL/g TS methane yield in control: 10.4 NmL/g TS	[90]
*Trichoderma reesei*	methane yield: 214 L/kg VS methane yield in control: 127 L/kg VS	[89]

## Data Availability

No new data were created or analysed in this study. Data sharing is not applicable to this article.
